# Anticipatory care planning for community-dwelling older adults at risk of functional decline: a feasibility cluster randomized controlled trial

**DOI:** 10.1186/s12877-022-03128-x

**Published:** 2022-05-25

**Authors:** Kevin Brazil, Christopher Cardwell, Gillian Carter, Mike Clarke, Dagmar Anna S. Corry, Tom Fahey, Patrick Gillespie, Anna Hobbins, Kieran McGlade, Peter O’Halloran, Nina O’Neill, Emma Wallace, Frank Doyle

**Affiliations:** 1 School of Nursing and Midwifery, Queen’s University Belfast, Belfast, Northern Ireland UK; 2 Centre for Public Health, ICSB, Royal Victoria Hospital, Queen’s University, Belfast, UK; 3 Department of General Practice, RCSI University of Medicine and Health Sciences, Dublin, Republic of Ireland; 4 Health Economics and Policy Analysis Centre, Institute for Lifecourse and Society, National University of Ireland, Galway (NUI Galway), Republic of Ireland; 5 School of Medicine, Dentistry, and Biomedical Sciences, Dunluce Health Centre, Queen’s University Belfast, Belfast, Northern Ireland UK; 6 School of Social Sciences, Education and Social Work, Queen’s University Belfast, Belfast, Northern Ireland UK; 7 Department of Health Psychology, RCSI University of Medicine and Health Sciences, Dublin, Republic of Ireland

**Keywords:** Feasibility study, Anticipatory care planning, Primary care, Frailty, Functional decline, Costs

## Abstract

**Objectives:**

To determine the feasibility, implementation and outcomes of an Anticipatory Care Planning (ACP) intervention in primary care to assist older adults at risk of functional decline by developing a personalized support plan.

**Design:**

Feasibility cluster randomized control trial.

**Setting and participants:**

Eight primary care practices (four in Northern Ireland, United Kingdom and four in the Republic of Ireland) were randomly assigned to either intervention or control arm. Eligible patients were those identified in each practice as 70 years of age or older and assessed as at risk of functional decline. Study participants (intervention *n* = 34, control *n* = 31) and research staff were not blinded to group assignment.

**Anticipatory care intervention:**

The intervention delivered by a registered nurse including: a) a home-based patient assessment; b) care planning on the basis of a holistic patient assessment, and c) documentation of a support plan.

**Outcome measures:**

A conceptual framework (RE-AIM) guided the assessment on the potential impact of the ACP intervention on patient quality of life, mental health, healthcare utilisation, costs, perception of person-centred care, and reduction of potentially inappropriate prescribing. Data were collected at baseline and at 10 weeks and six months following delivery of the intervention.

**Results:**

All pre-specified feasibility indicators were met**.** Patients were unanimous in the acceptance of the ACP intervention. Health care providers viewed the ACP intervention as feasible to implement in routine clinical practice with attending community supports. While there were no significant differences on the primary outcomes (EQ-5D-5L: -0.07 (-0.17, 0.04) *p* = .180; CES-D: 1.2 (-2.5, 4.8) *p* = .468) and most secondary measures, ancillary analysis on social support showed responsiveness to the intervention. Incremental cost analysis revealed a mean reduction in costs of €320 per patient (95% CI -31 to 25; *p* = 0.82) for intervention relative to the control.

**Conclusions:**

We successfully tested the ACP intervention in primary care settings and have shown that it is feasible to implement. The ACP intervention deserves further testing in a definitive trial to determine whether its implementation would lead to better outcomes or reduced costs.

**Trial registration:**

Clinicaltrials.gov, ID: NCT03902743. Registered on 4 April 2019.

**Supplementary Information:**

The online version contains supplementary material available at 10.1186/s12877-022-03128-x.

## Background

As the population of older adults increases internationally, those who reside in the community require ever more complex care which can be challenging for the patients, family carers, general practitioners, and community agencies [1–3].

These challenges call for the development and evaluation of practical and cost-effective approaches to care for older adults. Initiating the management of long-term conditions in a timely fashion, before catastrophic and costly events, is a priority. Therefore, a number of models of ‘integrated care’, including outpatient and community care models, have been proposed to facilitate these needs [4]. Two successful US models include the Program of All-inclusive Care of the Elderly (PACE) and the Geriatric Resources for Assessment and Care of Elders (GRACE) model, which include comprehensive multidisciplinary teams working with a patient’s primary care physician [5, 6]. However, these approaches differ in terms of location of provision of care (homecare [GRACE] versus day health centres [PACE]), but also in terms of adoption of either standardised protocols (GRACE) or more personalised care (PACE).While these care models have been successful in the US context, it is unclear if they would transition to a different health setting. In addition, other models of care, that are perhaps more flexible and person-centred, and do not follow such standardised protocols, could be envisaged.

Anticipatory care planning (ACP), which is similar to other integrated care approaches, is designed to anticipate, avert, or delay future functional decline through early identification of at-risk individuals [7]. It may include considerations on health improvement and staying well. Personalized care is a central facet of anticipatory care, describing an agreed series of discussions between patient and health professional (maybe along with other professionals or family members) for the purpose of clarifying goals, choices and preferences, and to create an action plan based on this joint understanding [7]. It therefore combines the non-standardised personalised care plans similar to PACE, but delivers this at home, akin to the GRACE protocol. It is also distinctive from, but may include aspects of, advance care planning which usually has a palliative, end-of-life focus and is typically employed with established functional decline and usually older individuals who are already receiving regular personal care with their conditions and may be in a care home.

While there is promise in the role of preventive primary care interventions [8, 9], there is a necessity to test the ACP model in a robust manner to address a number of reported study limitations [8, 9]. The importance of doing this is increased on the island of Ireland by the provision of different care models in neighbouring jurisdictions, where people frequently avail of care and services across both sides of the border, and where interventions such as ACP may be implemented differently. We describe here a study to ascertain the feasibility of testing an ACP intervention in both jurisdictions found on the island of Ireland.

This study was designed to establish the feasibility of a full trial evaluating implementation and outcomes of a primary care based ACP intervention assisting older adults at risk for functional decline by creating a personalized support plan. Specific objectives were as follows:

1) Inform recruitment strategies and procedures for general practices and patients for a full trial;

2) Determine recruitment / retention rates and outcome variability to inform sample size calculations for a full trial;

3) Inform mechanisms for optimal intervention delivery and cluster trial procedures;

4) Assess patients’, their family carers’ and health care providers’ perception of the acceptability, appropriateness, benefits, and convenience of the ACP intervention;

5) Determine outcome measures and economic assessment strategies for a full trial.

## Methods

### Trial Design

A feasibility cluster randomized controlled (cRCT) trial was conducted. Eight general practitioner (GP) practices were chosen by convenience and later randomly assigned to either the intervention or usual care group (four per group) respectively before the screening of patients for enrolment. Practices were stratified by country (Northern Ireland (NI) and Republic of Ireland (RoI)) before randomisation. Following Guidelines for Reporting Involvement of Patients and the Public (GRIPP2) [12] we also engaged three members of the public to consult at routine project team meetings and to consider progress, next steps, and to advise on research documents. The trial protocol has been described in detail elsewhere [10], and we report the findings here according to CONSORT criteria [11] (Additional Files 1 and 2).

### Setting

The trial was conducted in two countries, NI and the RoI, who have different healthcare systems but share a border. NI is a region within the United Kingdom that provides a model of care under the National Health Service (NHS) free to the patient at point of delivery, whereas the RoI has a mixed public–private healthcare system. However, for those aged 70 and older, or those with low incomes, a General Medical Services (GMS) scheme allows free access to primary and most other health services.

### Participants, screening, and enrolment

We aimed to enrol 64 patients (32 per randomized group), with eight patients per GP practice. The inclusion criteria have been reported in detail in the protocol paper [10] with the exception of individuals residing in assisted living, who were initially excluded but were ultimately deemed eligible for participation, as the conditions were similar to individuals living within their own home. Therefore, people meeting the following criteria were enrolled: aged 70 or older; enrolled in GMS/NHS primary care; multimorbidity (defined as at least two chronic medical conditions); taking at least 4 regular medications; ability to complete questionnaires in English language. Receipt of palliative care, cognitive impairment (Mini-Mental State Examination score of 20 or less), psychosis, homelessness or long-term inpatient or nursing home care were exclusion criteria.

Participating GPs drew a sample of patients from their registry and selected patients who met the study criteria who they contacted by post with information about the project and inviting them to complete the PRISMA 7 questionnaire [13]. The PRISMA 7 is a best-practice instrument used for screening patients at-risk for frailty. A score of > 2 indicates risk of functional decline [14, 15], and those patients were subsequently eligible for enrolment in the study.

Study nurses, study pharmacist, GP practice staff and regional key health professionals were interviewed after completion of the implementation of the ACP intervention to ascertain feasibility outcomes.

### The intervention group

Full details about patient screening and enrolment, nurse training, intervention and usual care group, data collection, patient standardised interview, and measures are provided in the protocol paper [10], with essential summary information outlined here.

Study nurses from both NI and RoI completed a 3-day training programme which included study procedures; standards and practice of personalized care; using the Easy-Care Assessment [16]; and carrying out a medication review aided by a pharmacist. Study nurses were employed by the project and were not affiliated with the participating GP practises.

To initiate delivery of the ACP intervention, the study nurse first liaised with the patient’s GP practice to obtain a medical summary, then organized a home visit to complete a structured patient assessment that recorded patient social and health concerns. The home visits emphasised a personalized care style, encouraging dialogue with the patient and, if desired, a family carer, about current and future care needs and personal goals to facilitate the design of a person-centred care plan.

After the first home visit, the study nurse provided the patient’s medication list to the study pharmacist who conducted a desk-based medication review founded on recognized guidelines [17]. The medication review included, when needed, a telephone consultation between the study nurse and study pharmacist. The study nurse drafted a defined summative report of the assessment inclusive of patient goals, care preferences, identified challenges, and an action list. She informed the patient’s GP of the patient assessment results and the GP recommended actions, provided feedback, and confirmed the suggested care plan. After the GP consultation, and depending on the difficulty of identified care needs, the study nurse either met with the patient again or contacted them by telephone. Through this meeting the patient’s identified priorities and identified options for support were confirmed and discussed. A full description of the intervention, according to the TIDieR [18] checklist is provided in Additional File 3.

### Usual care group

Patients in the usual care group provided with standard care, meaning they could request appointments with their GPs to discuss any health problems as they arose. This comparator was viewed as reactive rather than anticipatory care.

### Outcomes and data collection

The RE-AIM (reach, effectiveness, adoption, implementation, maintenance) framework guided our approach to evaluating the ACP intervention [19–21]. Four of the five parameters of the framework apply to this study. A fifth, maintenance, was not considered as it examines integration into routine practice post-implementation.

Quantitative evaluation of reach and effectiveness**:** Quantification of reach concerned characteristics of the study sample and their representativeness. Baseline variables included demographics such as age, gender, education, living arrangements, income, and economic resources.

Effectiveness pertained to the primary and secondary outcomes of the trial. Patients in the trial participated in standardised individual interviews at baseline, ten weeks, six months post intervention, with six months being the primary outcome time point. These interviews were conducted by researchers, not the interventionist nurses.

Primary outcome measures were the EQ-5D-5L, which assesses quality of life [22]; and the Center for Epidemiological Studies Depression Scale (CES-D) [23], a widely used depression screening measure.

Secondary outcome measures included the Katz Index of Independence in Activities of Daily living [24] which examines functioning; the Generalised Anxiety Disorder 7 (GAD-7) [25]; and the Patient Assessment of Chronic Illness Care (PACIC) Scale where patients rate their satisfaction with their care [26].

Ancillary outcome: The Medical Outcomes Study (MOS) Social Support Survey [27] was initially intended as a baseline measure only, but we hypothesised that changes due to the ACP intervention might be observed and it was later decided to include this measure at all three measurement points.

Health economics analysis was conducted to provide preliminary estimates of the costs, outcomes, and potential cost effectiveness of the ACP intervention compared to usual care over a six-month follow up period. The healthcare provider perspective was adopted with regard to costing, and health outcomes were stated in terms of quality adjusted life years (QALYs, based on the EQ-5DL-5L [28–30]). Participant responses to structured questionnaires provided data on resource use and health status. Two cost elements were included in the cost analysis, both expressed in Euros (€) and Sterling (£) in 2019 prices. First, the implementation costs and the ACP intervention were estimated for the RoI and NI. Second, costs regarding the use of primary and secondary healthcare services over the course of the follow up period were calculated. The Irish EQ-5D-5L value set [31] was applied to generate the utility values, with area under the curve methods employed to generate the QALYs over the follow up period of six-months [32]. Incremental cost and QALYs analyses were conducted to compare alternatives.

Medication management: a medication review by the study pharmacist was performed after liaison with the relevant study nurse. Mean number of changes to any prescribed medications were ascertained.

Qualitative evaluation of adoption and implementation: Adoption examined patient and provider acceptability of the ACP intervention. Patient acceptability of the intervention was assessed using a semi-structured topic guide at 10-week follow-up for participants in the intervention group. Questions assessed patients’ views on the intervention overall and its constituent parts, its implementation, and suggestions for refining the intervention. Study nurses, the pharmacist, GPs, and GP Practice Managers were interviewed on completion of the intervention. Interviews covered aspects of the intervention including necessary qualifications of the nurse; training needs; building rapport with participants; suitability of the home setting for patient interactions; and the potential benefits of the ACP intervention situated in the GP practice. Key health professional interviews were also conducted to identify the factors that could influence how the ACP intervention could integrated into the regional care systems in the future.

Pre-specified criteria for proceeding to full trial were as follows: acceptability to 70% or more of patients, carers’, and staff; intervention is perceived by staff to be implementable; recruitment of at least 50% of eligible patients; retention of at least 65%of recruited patients; potentially detectable differences in primary and secondary outcomes; and feasible ascertainment of the needed economic evaluation variables.

### Sample size

We aimed for 64 patients (32 in each randomized arm) to allow for calculation of the standard deviation of the EQ-5D-5L.

### Randomisation

GP practices were ordered alphabetically by jurisdiction and urban/rural status and allocated a number. The random number function in Microsoft Excel was used by the blinded study statistician to assign random numbers to the practices. The lowest number urban and rural practice by jurisdiction were then assigned to the intervention group. Subsequently, the study nurses were informed of the GP practice allocation and informed the practices. Study participants and research staff were not blinded to group assignment.

### Quantitative analysis

Summary statistics (mean and standard deviations) and frequencies/proportions were used to summarize variables at baseline, with standardised differences between intervention and usual care groups calculated for these variables. The ACP intervention and control group were formally compared, recognising that no prior sample size was conducted and that these analyses were likely not powered for detecting expected effects. Analysis of covariance was used to calculate the mean difference (and 95% confidence interval) of outcomes at follow-up, comparing the intervention with the control group, adjusting for baseline scores, gender, age region, living arrangements, carer and cared for status in a complete case analysis [33]. Clustering at GP level was accounted for by using robust standard errors [34]. These analyses were repeated for data from week 10 and six months. We also supply other supplementary analyses, including models that were adjusted for other potential baseline differences; multilevel models instead of robust standard errors; and models using multiple imputation.

Mann–Whitney U-test was used to assess differences between the intervention and usual care groups on changes to prescribed medications. For the health economic analysis, the ACP intervention and usual care was compared on the basis of a statistical analysis of the incremental costs and incremental QALYs at follow up, estimated using all complete cases.

All participants were analysed according to the intention-to-treat (ITT) principle; that individuals remained in the intervention group regardless of whether or not they engaged with the intervention. All statistical analyses were conducted in Stata v16.

### Qualitative analysis

NVivo-12, QSR International [35], was utilised in the organisation and analysis of qualitative results. The template analysis style, outlined previously [36], was used for analysis, with themes, patterns, and interrelationships identified and interpreted, leading to a theme structure and final thematic framework. More detail on qualitative analyses is available elsewhere [37, 38].

## Results

### Recruitment and retention

Recruitment of participants took place in RoI between March and May 2018 and then in NI between May and October 2018. The recruitment process ceased at the point at which the intended sample size was reached.

**Fig. 1:**
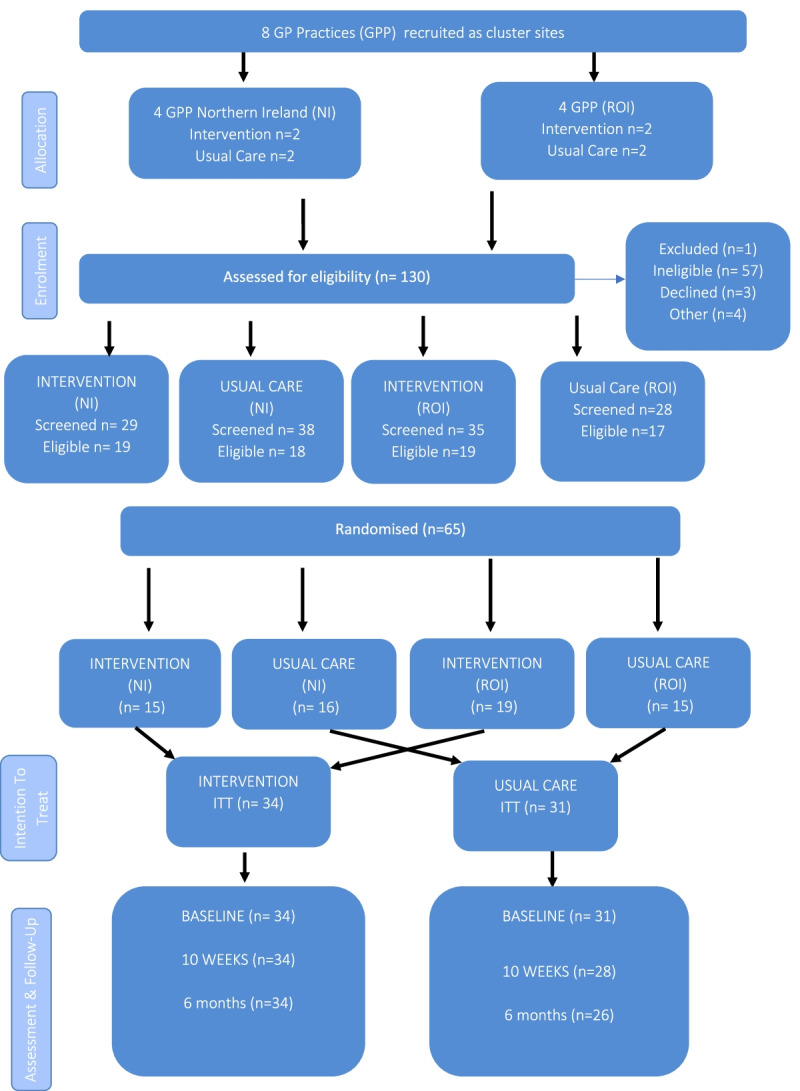
Participant Flow Diagram

Figure 1 illustrates that a total of eight GP practices were recruited to the trial across the island of Ireland. Four GP practices were recruited in Northern Ireland via the Northern Ireland Clinical Research Network and four were recruited in the Republic of Ireland by research team members.

A total of 130 patients were screened for eligibility, with 73 deemed eligible following return of the PRISMA-7 form. Eight of these eligible patients either did not consent to participate in the study or were withdrawn for other reasons. Five individuals did not complete the study; 1 participant died during the trial while the remaining 4 participants did not respond to follow-up correspondence (loss to follow-up).

### Intervention activities

The type of clinical concerns raised by patients at the study nurse visit included digestive problems, mobility problems, chest pain and breathlessness, sleep problems; dry skin problems, diabetes related issues, problems with hearing and vision, as well as problems with and queries about medications. The nurses identified actions which included immediate self-help advice, written information, referral to GP, discussions with GP and study pharmacist about the patient’s priority concerns, change in medications, and further referrals where appropriate.

The average time spent by the study nurse on a patient case was 441.28 min (SD = 106.43). This included travel, administration, consultation with the study pharmacist and GP practice. The nurses spent an average of 165.50 min (SD = 66.47) in direct patient contact. Face to face patient contact was an average 138.35 min (SD = 60.94) and an average 27.15 min (SD = 17.93) on telephone contact. The study pharmacist conducted an average of 7.8 (SD = 3.20) medication interventions per patient overall. This included 265 medication interventions in total (156 medication optimisation, 68 queries, 41 advice).

### Reach and Effectiveness

#### Baseline data

Table 1 shows the baseline demographics for participants in each randomized group. The distribution of age and gender was similar between the two groups. Living arrangements were also similar in both groups, with the exception of assisted living; no one from the intervention group reported assisted living, whilst 13% (4.31) from the usual care group did. Four people resided in housing (assisted living) where services were provided to support independent living. Almost twice as many participants from the intervention group (8/34, 23/5%) reported living with extended family compared to usual care group (4/31, 12.9%). Almost half of the usual care group (14/31, 45.2%) reported that someone provided care for them, in comparison with less than a third of the intervention group (10/34, 29.4%).

**Table 1:** Baseline characteristics

	**Intervention**	**Usual care**	**Standardized difference**
	*n* (%)	*n* (%)	
Age **[mean (SD)]**	34/34 (100.0%) [79.2 (5.4)]	31/31 (100.0%) [81.8 (5.7)]	-0.47
Gender			
Male	18/34 (52.9%)	16/31 (51.6%)	0.03
Female	16/34 (47.1%)	15/31 (48.4%)	-0.03
Region			
Northern Ireland	15/34 (44.1%)	16/31 (51.6%)	-0.15
Republic of Ireland	19/34 (55.9%)	15/31 (48.4%)	0.15
Living arrangements			
Alone	13/34 (38.2%)	11/31 (35.5%)	0.06
Couple	13/34 (38.2%)	12/31 (38.7%)	-0.01
With extended family	8/34 (23.5%)	4/31 (12.9%)	0.28
Assisted living	0/34 (0.0%)	4/31 (12.9%)	-0.54
Employment			
Full-time	1/34 (2.9%)	0/31 (0.0%)	0.25
Part-time	0/34 (0.0%)	1/31 (3.2%)	-0.26
Retired	33/34 (97.1%)	30/31 (96.8%)	0.02
Are you a carer for someone?			
No	29/34 (85.3%)	28/31 (90.3%)	-0.15
Yes	5/34 (14.7%)	3/31 (9.7%)	0.15
Does someone provide care for you?			
No	24/34 (70.6%)	17/31 (54.8%)	0.33
Yes	10/34 (29.4%)	14/31 (45.2%)	-0.33

Note: Standardized mean difference for age between groups was 0.5

### Primary and secondary outcomes

Table 2 presents data on all measures at baseline compared with 10 weeks and six months. Comparison between baseline and each follow-up time-point are given but it is recognised that the analyses are underpowered for definitive tests of significance.

**Table 2:** Comparisons of outcomes between intervention and usual care at 10 weeks and 6 months based upon complete case, adjusting for gender, age, region, living arrangements, carer and cared for

**Outcome**	**Intervention**				**Usual care**					**Diff in mean**^**1**^ **(95% CI)**	***P***	**Adjusted**^**2**^** diff in mean (95% CI)**	***P***
	Baseline		Endpoint		Baseline			Endpoint					
	*N*	Mean (sd)	*N*	Mean (sd)	*n*	Mean (sd)	*n*		Mean (sd)				
**10 week analysis**													
**Primary outcomes**													
EQ-5D-5L index score	34	0.72 (0.19)	34	0.69 (0.21)	31	0.65 (0.25)	28		0.65 (0.31)	-0.03 (-0.17,0.12)	0.668	-0.01 (-0.07,0.05)	0.712
EQ-VAS score	34	62.2 (19.9)	34	64.9 (15.4)	31	61.2 (15.6)	28		65.9 (18.2)	-1.4 (-8.8,6.0)	0.676	-1.4 (-9.1,6.2)	0.671
CES-D	34	9.1 (9.1)	34	9.3 (9.6)	31	10.6 (9.1)	28		8.5 (8.8)	2.1 (-1.0,5.3)	0.154	2.4 (-0.5,5.2)	0.091
**Secondary outcomes**													
PACIC	34	2.0 (0.5)	34	2.1 (0.8)	31	2.1 (0.7)	28		1.8 (0.5)	0.3 (-0.2,0.9)	0.174	0.4 (-0.0,0.7)	0.053
KATZ Index	34	5.4 (1.0)	34	5.3 (0.8)	31	5.1 (1.3)	28		5.2 (1.1)	-0.1 (-0.4,0.1)	0.168	-0.2 (-0.5,0.1)	0.105
GAD-7	34	2.3 (3.2)	34	2.6 (3.2)	31	2.5 (2.8)	28		2.4 (2.7)	0.4 (-1.3,2.1)	0.574	0.1 (-1.4,1.5)	0.929
MOS Social Support Score	34	4.2 (0.8)	34	4.5 (0.5)	31	4.3 (0.8)	28		4.2 (1.1)	0.4 (-0.1,0.8)	0.076	0.5 (-0.0,1.0)	0.056
**6 month analysis**													
**Primary outcomes**													
EQ-5D-5L index score	34	0.72 (0.19)	34	0.65 (0.27)	31	0.65 (0.25)	26		0.67 (0.28)	-0.07 (-0.22,0.08)	0.316	-0.07 (-0.16,0.03)	0.139
EQ-VAS score	34	62.2 (19.9)	34	63.1 (20.2)	31	61.2 (15.6)	26		66.9 (12.3)	-4.0 (-13.5,5.5)	0.352	-5.1 (-15.6,5.5)	0.292
CES-D	34	9.1 (9.1)	34	9.6 (7.1)	31	10.6 (9.1)	26		8.4 (7.7)	1.6 (-2.1,5.3)	0.341	1.2 (-1.2,3.6)	0.29
**Secondary outcomes**													
PACIC	34	2.0 (0.5)	34	2.1 (0.9)	31	2.1 (0.7)	26		1.8 (0.8)	0.5 (0.0,0.9)	0.048	0.4 (0.0,0.8)	0.049
KATZ Index	34	5.4 (1.0)	34	5.3 (1.2)	31	5.1 (1.3)	26		5.2 (1.2)	-0.2 (-0.8,0.3)	0.313	-0.3 (-0.5,0.0)	0.053
GAD-7	34	2.3 (3.2)	34	3.1 (3.5)	31	2.5 (2.8)	26		2.3 (3.3)	0.8 (-0.6,2.3)	0.227	0.3 (-1.2,1.9)	0.637
MOS Social Support Score	34	4.2 (0.8)	34	4.4 (0.7)	31	4.3 (0.8)	26		3.7 (1.1)	0.7 (0.2,1.3)	0.018	0.8 (0.4,1.2)	0.001

^1^ Using ANCOVA, and adjusting for clustering using robust standard errors (with 8 practices)

^2^ Same as ^1^ but additionally adjusting for gender, age, region, living arrangements (alone/couple/extended family/assisted living), carer (yes/no) and cared for (yes/no)

### Primary outcome measures

Table 2 reports scores on the EQ-5D-5L and CES-D for both groups at each time-point. The study did not find any statistically significant difference regarding the EQ-5D-5L and CES-D outcomes, for either the primary outcome time point at six months, or the earlier 10-week time point.

### Secondary outcome measures

Table 2 shows a small increase in the PACIC from baseline and 6-months in the intervention group, and a small decrease from baseline in the usual care group at both time-points. There is a statistically significant difference in the unadjusted analysis for the 6-month timepoint, albeit this did not survive full statistical adjustment.

The KATZ Index at baseline shows a high level of functioning for the ACP intervention and usual care groups. Table 2 reveals no significant difference between baseline and week 10 for the ACP intervention and usual care group. Similarly, there was no significant difference between baseline and six months on the GAD-7 was revealed between the ACP intervention group and usual care group. A Mann–Whitney U-Test showed that the total number of changes in medication from baseline to six months was not statistically significant, albeit a limitation of this test is that it does not account for clustering.

### Ancillary outcome

Participants from both arms of the study reported similar levels of social support on the Medical Outcomes Study (MOS) Social Support Survey at baseline. For those in the ACP intervention group Table 2 revealed a trend for an increase in perceived social support compared to the usual care group at 10 weeks, that was a statistically significant difference at six months, suggesting that patients who participated in the ACP intervention felt more supported after the intervention. For those in the usual care arm of the study, a perceived decrease in social support was noted between baseline and six months.

All results were similar when using the fully adjusted models, imputed models or modelling with random intercept instead of robust standard errors (see Supplementary Tables 1,2,3). One difference was that the MOS Social Support scores were higher in the intervention group based on multilevel modelling approaches (Supplemental Table 1). Supplementary Table 4 reports the interclass correlation coefficients (ICCs) for the primary outcomes.

### Health economics analysis

The results are presented in Tables 3, 4 and 5. Summary resource use data are presented in Table 3 and summary EQ-5D-5L data are presented in Table 4. The incremental cost and QALYs analyses are presented in Table 5. Unit cost estimates are presented in Additional file 4. The implementation cost of the ACP intervention was estimated at €769 per patient. The mean healthcare cost per patient was estimated at €2,518 (SD = 2,227) for the intervention group and €2,838 (SD: 5,569) for the control group. The incremental analysis revealed a mean reduction in costs of €320 per patient (95% CI -31 to 25; *p* = 0.82) for the intervention relative to the control. The mean QALYs at 6 months per patient was estimated at 0.34 (SD = 0.09) for the intervention group and 0.33 (SD = 0.14) for the control group. The incremental analysis revealed a mean increase in QALYs of 0.01 per patient (95% CI -0.04 to 0.06; *p* = 0.65) for the intervention relative to the control. An additional analysis, estimated controlling for baseline utility EQ-5D-5L scores, revealed a mean reduction in QALYs of 0.01 per patient (95% CI -0.05, 0.02; *p* = 0.423) for the intervention relative to the control. Neither the differences in mean costs or mean QALYs were statistically significant.

**Table 3:** Summary resource use data at baseline and follow Up by treatment arm

	**Intervention**	**Control**	**Intervention**	**Control**	**Intervention**	**Control**
	Baseline *Mean(SD)*		10 weeks *Mean(SD)*		6 months *Mean(SD)*	
**Resource Items**						
GP Visits	5.2(3.4)	4.6(2.6)	1.4(1.5)	1.3(1.7)	2.5(2.0)	2.0(1.8)
Practice Nurse visits	2.6(2.6)	2.8(2.6)	0.8(1.3)	0.8(0.7)	1.2(1.7)	0.8(1.1)
Public Health/District Nurse visits	0.3(1.4)	1.1(4.9)	0.2(0.5)	0.0(0.2)	0.1(0.5)	0.8(3.3)
Specialist Nurse visits	0.4(0.6)	0.5(1.4)	0.1(0.4)	0.0(0.2)	0.3(0.6)	0.2(0.4)
Chiropody visits	0.9(1.9)	0.9(1.6)	0.4(0.6)	0.3(0.5)	0.9(1.1)	0.7(1.2)
Physiotherapy visits	1.4(2.8)	1.9(3.6)	0.6(1.6)	0.4(0.9)	0.8(1.8)	1.3(3.1)
Occupational Therapist visits	0.1(0.3)	0.2(0.5)	0.1(0.6)	0.1(0.4)	0.1(0.4)	0.2(0.5)
Optician visits	1.1(0.6)	1.3(1.2)	0.3(0.5)	0.5(0.6)	0.7(1.0)	0.3(0.6)
Social Worker visits	0.0(0.0)	0.1(0.4)	0.0(0.2)	0.0(0.2)	0.0(0.0)	0.0(0.2)
Psychological Services visits	0.0(0.0)	0.1(0.5)	0.0(0.0)	0.0(0.0)	0.1(0.3)	0.0(0.0)
Other Services visits	0.1(0.4)	0.3(0.9)	0.2(0.4)	0.1(0.4)	0.3(0.7)	0.0(0.2)
Day Care visits	0.0(0.0)	1.5(5.5)	0.2(0.7)	0.0(0.2)	0.1(0.2)	0.2(0.6)
Outpatient Visits	0.8(0.4)	0.7(0.4)	0.4(0.5)	0.7(0.5)	0.6(0.5)	0.5(0.5)
Inpatient Days	0.0(0.0)	1.9(10.0)	0.0(0.2)	0.0(0.0)	0.2(0.5)	0.0(0.2)
Inpatient Nights	6.4(26.5)	2.0(4.0)	0.8(2.7)	0.3(1.0)	0.6(2.0)	2.0(5.7)
A&E Visits	0.6(0.9)	0.7(0.9)	0.1(0.4)	0.2(0.5)	0.2(0.5)	0.3(0.7)

**Table 4:** Summary EQ-5D-5L domain data at baseline and follow-up by treatment arm

		**Intervention**		**Control**	
**Dimensions**	**Levels**	**Baseline**	**Follow-up**	**Baseline**	**Follow-up**
			**(6 months)**		**(6 months)**
		*N *= 34	*N* = 34	*N* = 31	*N* = 26
		*N* (%)	*N* (%)	*N* (%)	*N* (%)
**Mobility**					
	**None**	7(20.59)	4(11.76)	6(19.35)	5(19.23)
	**Slight**	11(32.35)	15(44.12)	10(32.26)	10(38.46)
	**Moderate**	14(41.18)	9(26.47)	9(29.03)	5(19.23)
	**Severe**	2(5.88)	6(17.65)	4(12.90)	6(23.00)
	**Unable**	0.00	0.00	2(6.45)	0.00
**Self-care**					
	**None**	27(79.41)	24(70.59)	22(70.97)	13(50.00)
	**Slight**	2(5.88)	5(14.71)	5(16.13)	4(15.38)
	**Moderate**	5(14.71)	4(11.76)	1(3.23)	4(15.38)
	**Severe**	0.00	1((2.94)	1(3.23)	3(11.54)
	**Unable**	0.00	0.00	2(6.45)	2(7.69)
**Usual activities**					
	**None**	11(32.35)	11(32.35)	14(45.16)	8(30.77)
	**Slight**	14(41.18)	13(38.24)	3(9.68)	6(23.08)
	**Moderate**	5(14.71)	5(14.71)	8(25.81)	5(19.23)
	**Severe**	3(8.82)	5(14.71)	1(3.23)	3(11.54)
	**Unable**	1(2.94)	0.00	5(16.13)	4(15.38)
**Pain/Discomfort**					
	**None**	7(20.59)	5(14.71)	10(32.26)	11(42.31)
	**Slight**	14(41.18)	11(32.35)	8(25.81)	9(34.62)
	**Moderate**	11(32.35)	13(38.24)	12(38.71)	6(23.08)
	**Severe**	2(5.88)	3(8.82)	1(3.23)	0.00
	**Extreme**	0.00	2(5.88)	0.00	0.00
**Anxiety/Depression**					
	**None**	18(52.94)	18(52.94)	12(38.71)	16(61.54)
	**Slight**	12(35.29)	11(32.35)	12(38.71)	5(19.23)
	**Moderate**	3(8.82)	4(11.76)	6(19.35)	5(19.23)
	**Severe**	1(2.94)	1(2.94)	1(3.23)	0.00
	**Extreme**	0.00	0.00	0.00	0.00

**Table 5:** Incremental cost and QALY analysis at follow up

	**Intervention**	**Control**
**Cost**	**Mean (SD)**	**Mean (SD)**
Healthcare Cost	1,749(2,226)	2,838(5,569)
ACP CHITIN Programme	769(0)	0 (0)
Total Cost	2,518(2,227)	2,838(5,569)
Incremental Cost (95% CI) (p-value)	-320 (-3102,2463) [0.822]	
Health Outcomes	Mean (SD)	Mean (SD)
EQ-5D-5L Index Score at baseline	0.72(0.19)	0.65(0.25)
EQ-5D-5L Index Score at 3 months	0.69(0.21)	0.65(0.31)
EQ-5D-5L Index Score at 6 months	0.65(0.27)	0.65(0.31)
QALYs Gained	0.34(0.10)	0.33(0.14)

### Implementation and adoption of the ACP intervention

Thirty-four patients (RoI = 19, NI = 15) from the ACP intervention group completed qualitative interviews at 10 weeks. The results of these interviews are reported in more detail in another paper and are summarised here [37]. Patients interviewed reported unanimous acceptance of the ACP intervention, as well as its individual components. Anchoring the ACP intervention in the GP practice was crucial for both successful patient recruitment, and acceptability of the ACP intervention. Home visits by the study nurse were fully appreciated by participating patients and regarded as very beneficial and convenient. The person-centred approach taken in the patient assessment by our nurses was essential to building rapport with the patients and their acceptability of the intervention. The medication review conducted by the study pharmacist was seen as a very helpful component of the intervention.

Twelve interviews were conducted (RoI = 5, NI = 7) with individuals closely associated with the implementation of the ACP intervention. Study nurses (*N* = 5) endorsed the home visits as ideal for the ACP intervention, together with anchoring the intervention in the GP practice. Standardised protocols including improved patient selection for participation in the intervention, standardised assessment tools, and effective communication between the study nurses and GP practice were highlighted as valuable implementation facilitators. Stakeholders located in GP practices (GPs = 3, Practice managers = 3) viewed the nurse-led intervention favourably, with the caveat that existing efforts like medical care planning and comprehensive geriatric assessment are not duplicated. The study pharmacist (*N* = 1) highlighted differences in handling data protection guidelines between practices as a barrier for access to patient records.

Sixteen (RoI = 7, NI = 9) individuals participated in the key health professional interviews. These included individuals with managerial roles in public health agencies, voluntary sector, and care homes; geriatricians, public health nurses and allied health professionals. There was consensus that the ACP intervention is best placed in a general practice setting, delivered by a specifically trained nurse, working in partnership with an integrated, multidisciplinary team. Significant long-term benefits were said to include the potential to avoid hospital admissions and further frailty, increasing and extending independence and quality of life, avoiding reactive care and crisis management through preventative care. An identified pathway, use of existing structures, shared medical records, a standardized approach, and specific person-centred training for nurses were among the recommendations for successful implementation. These key informants also indicated that effectiveness and sustainability of the ACP intervention will depend on removing or circumnavigating systemic barriers to sustain an ACP service within the existing systems. Notwithstanding the known barriers, the favourable views reported by community key informants suggest that a full-scale randomized controlled trial is warranted in order to provide much-needed evidence and impetus for such change.

## Discussion

This feasibility study has shown that several necessary conditions for the development of a definitive study has been met. These included that the ACP intervention was acceptable to > 70% patients and health care professionals; it can be readily implemented (> 35% GP practices, > 50% patients) and that two-thirds of the recruited patients were retained in the study. Progression criteria also included the presence of a potentially detectable change in primary and secondary outcomes. As expected, the small sample in the feasibility study meant that statistically significant changes were not found in the primary outcomes, but significant differences were found in the MOS social support scale. This revealed that patients who participated in the ACP intervention experienced stronger social support compared to usual care participants. Finally, the findings from the health economic analysis highlight the feasibility of conducting a health economic evaluation alongside a definitive trial to answer questions of cost effectiveness.

Our preliminary results show that the ACP intervention has the potential to be cost effective; but further research is clearly required to address this question directly. Ploeg et al. [8] conducted a meta-analysis on the effectiveness of preventive primary care outreach interventions aimed at older people. The authors assessed the quality of studies and extracted information on 19 trials involving 14,911 patients. The review showed that studies of preventive care outreach interventions aimed at older people were associated with a 17% reduction of mortality and a 23% increased likelihood of continuing to live in the community. A recent Cochrane review conducted by Susan Smith and colleagues [9] investigated the impact of interventions to improve the outcomes of patients with multi-morbidity in primary care. Ten studies including 3407 patients examining a range of interventions demonstrated mixed effects. The authors of both studies identified a number of shortcoming that including failure to include quality of life and cost-effectiveness as outcome measures, consideration of potential lack of statistical power to detect clinically important differences between groups, reduction of potentially inappropriate prescribing, the use of screening tools that lacked demonstrated predictive validity, and poorly described interventions. The results of the feasibility study go some way to addressing many of these limitations. Specifically, we demonstrated both the suitability of the research design, selection of outcomes measures and user acceptability of the ACP intervention for a future clinical trial that offers the opportunity to address identified limitations of research conducted to date. A lesson learned from this study for a future trial pertains to the selection of primary outcomes. Primary outcomes may be represented with the use of the EQ-5D-5L which is needed for costs and the inclusion of a QoL measure. The MOS Social support measure also holds promise as a primary outcome. Depression (CES-D) could be relegated to a secondary outcome.

This ACP project was developed in an integrated cross-border fashion across NI and the RoI. The study nurses employed in both jurisdictions were trained together. General practices, patients, and key health professionals were recruited in both jurisdictions and data collected were combined for analyses with consideration to comparative jurisdictional difference. Data quality, standards and security had to be set out in standard procedures that adhered to both jurisdictions.

## Conclusions

We have successfully tested the ACP intervention in primary care settings and shown that it was both acceptable by patients and perceived by health care providers as feasible to implement in normal clinical practice. We have shown that the ACP intervention deserves further testing in a definitive trial to determine if its implementation would lead to better outcomes for patients.

## Disclaimer

The views and opinions expressed in this paper do not necessarily reflect those of the European Commission or the Special EU Programmes Body (SEUPB).

## Completeness of data

### Intervention

Baseline- 0% missing data for GP visits, public health/district nurse visits , specialist nurse visits , chiropody visits, physiotherapy visits, occupational therapist visits, optician visits , social worker visits, other services visits, and EQ-5D-5L. 2.94% missing data for practice nurse visits, 2.94% missing data for psychological services visits, 8.82% missing data for day care visits, 2.94% missing data for outpatient visits 2.94% missing data for inpatient days and 2.94% missing data for inpatient nights, and 2.94% missing data for A&E visits

## Intervention

Follow-up (6 months)- 0% missing data for GP visits, practice nurse visits, public health/district nurse visits, specialist nurse visits, chiropody visits, physiotherapy visits, occupational therapist visits, optician visits, social worker visits, psychological services visits, other services visits, day care visits, outpatient visits, inpatient days, inpatient nights, A&E visits and EQ-5D-5L.

## Control

Baseline –—0% missing data for GP visits, public health/district nurse visits, specialist nurse visits, chiropody visits, physiotherapy visits, occupational therapist visits, optician visits, social worker visits, psychological services visits, other services visits, day care visits, outpatient visits, inpatient days, inpatient nights and EQ-5D-5L. 3.23% missing data on practice nurse visits, 3.23% missing on other services visits, 6.45% missing on A&E visits

## Control

Follow-up (6 months)- 16.13% missing data for GP visits, 16.13% missing data for practice nurse visits, 16.13% missing data for public health/district nurse visits, 16.13% missing data for specialist nurse visits, 22.58% missing data for chiropody visits, 16.13% missing data for physiotherapy visits, 16.13% missing data for occupational therapist visits, 16.13% missing data for optician visits, 16.13% missing data for social worker visits, 16.13% missing data for psychological services visits , 16.13% missing data for other services visits, 16.13% missing data for outpatient visits, 16.13% missing data for inpatient days and 16.13% missing data for inpatient nights, 16.13% missing data for A&E visits and 16.13% missing data for EQ-5D-5L.

## Supplementary Information

**Additional file 1.**CONSORT Checklist

**Additional file 2.** CONSORT Checklist extension for abstracts

**Additional file 3.** TIDieR Checklist 

**Additional file 4.** Unit cost estimates in 2019 prices

**Additional file 5: Supplementary Table 1.** Comparisons of outcomes between intervention and usual care at 10 weeks and 6 months, based upon complete case using a multilevel model to calculate difference in mean and adjusted difference in mean.

**Additional file 6: Supplementary Table 2.** Comparisons of outcomes between intervention and usual care at 10 weeks and 6 months, based upon multiple imputation.

**Additional file 7: Supplementary Table 3.** Medians and interquartile ranges of outcomes at 10 weeks and 6 months.

**Additional file 8: Supplementary Table 4.** ICCs for primary outcomes

## Data Availability

The quantitative data resulting from this study can be found in Clinicaltrials.gov, ID: NCT03902743. The qualitative data underlying this study cannot be shared publicly because they are qualitative patient interviews which contain personal and potentially identifiable information, and participants have consented to publication of anonymous quotes only. Requests for data can be made to Queen’s University Belfast Research Governance, Ethics and Integrity department (researchgovernance@qub.ac.uk) with appropriate ethical approval.

## Abbreviations

ACP: Anticipatory Care Planning
CES-D: Center for Epidemiological Studies Depression Scale
EQ-5D-5L EuroQoL: Quality of Life instrument
GAD-7: General Anxiety Disorder screening tool
GP: General Practitioner
ITT: Intention to Treat
KATZ: Katz Index of Independence in Activities of Daily living
MOS: Medical Outcomes Study Social Support Survey
NI: Northern Ireland
PACIC: Patient Assessment of Chronic Illness Care
QALYS: Quality Adjusted Life Years
ROI: Republic of Ireland
