# Supplementary material for: Anticipatory care planning for community-dwelling older adults at risk of functional decline: a feasibility cluster randomized controlled trial
Source: BMC Geriatr. 2022 May 25;22:452. doi: 10.1186/s12877-022-03128-x (PMC9131621; doi:10.1186/s12877-022-03128-x)
Supplement: Supplementary file 2 — Additional file 2. CONSORT Checklist extension for abstracts [file 12877_2022_3128_MOESM2_ESM.doc]

**Additional file 2 - CONSORT Checklist Extension for Abstracts:**

**Items to include when reporting a randomized trial in a journal or conference abstract**

| **Item** | **Description** | **Reported on line number** |
| --- | --- | --- |
| Title | Identification of the study as randomized | 1-3 |
| Authors * | Contact details for the corresponding author | N/A |
| Trial design | Description of the trial design (e.g. parallel, cluster, non-inferiority) | 45 |
| Methods |  |  |
| Participants | Eligibility criteria for participants and the settings where the data were collected | 46-48 |
| Interventions | Interventions intended for each group | 49-51 |
| Objective | Specific objective or hypothesis | 41-44 |
| Outcome | Clearly defined primary outcome for this report | 52-56 |
| Randomization | How participants were allocated to interventions | 45-48 |
| Blinding (masking) | Whether or not participants, care givers, and those assessing the outcomes were blinded to group assignment | 49 |
| Results |  |  |
| Numbers randomized | Number of participants randomized to each group | 49 |
| Recruitment | Trial status |  |
| Numbers analysed | Number of participants analysed in each group | 60-61 |
| Outcome | For the primary outcome, a result for each group and the estimated effect size and its precision | 62-63 |
| Harms | Important adverse events or side effects | None |
| Conclusions | General interpretation of the results | 57-67 |
| Trial registration | Registration number and name of trial register | 69-70 |
| Funding | Source of funding | 71-75 |

**this item is specific to conference abstracts*
