# Supplementary material for: Anticipatory care planning for community-dwelling older adults at risk of functional decline: a feasibility cluster randomized controlled trial
Source: BMC Geriatr. 2022 May 25;22:452. doi: 10.1186/s12877-022-03128-x (PMC9131621; doi:10.1186/s12877-022-03128-x)
Supplement: Supplementary file 4 — Additional file 4. Unit cost estimates in 2019 prices [file 12877_2022_3128_MOESM4_ESM.docx]

**Additional file 4: Unit Cost Estimates in 2019 Prices**

|  |  | **Republic of Ireland** | | **Northern Ireland** | | **STG £ converted to € euro using PPP 2019** |
| --- | --- | --- | --- | --- | --- | --- |
| **Healthcare Resources** | **Activity** | **Unit Cost €** | **Source** | **Unit Cost £** | **Source** |  |
| GP Visits: | Per Visit | €60 | Study Records | £39 | PSSRU 2019 | €46 |
| Practice Nurse Visits | Per Visit | €40 | Study Records | £8 | PSSRU 2019 | €9 |
| Public Health  /District Nurse | Per Visit 1hour | €48 | HSE | £46 | PSSRU 2019 | €54 |
| Specialist Nurse | Per Visit 1hour | €49 | HSE | £46 | PSSRU 2019 | €54 |
| Chiropody | Per Visit 30 min. | €22 | HSE | £17 | PSSRU 2019 | €20 |
| Physiotherapy | Per Visit 30 min | €22 | HSE | £17 | PSSRU 2019 | €20 |
| Occupational Therapist | Per Visit 1hour | €44 | HSE | £44 | PSSRU 2019 | €52 |
| Optician | Per Visit 30 min | €22 | HSE | £17 | PSSRU 2019 | €20 |
| Social Worker | Per Visit 1hour | €44 | HSE | £45 | PSSRU 2019 | €53 |
| Psychological Services | Per Visit 1hour | €58 | HSE | £54 | PSSRU 2019 | €63 |
| Day Care | Per Visit | €38 | (Brick et al., 2017) | £45 | PSSRU 2019 | €53 |
| Outpatient Visits | Per Visit | €136 | HPO | £136 | PSSRU 2019 | €159 |
| Inpatient Days | Per Day | €665 | HPO | £757 | PSSRU 2019 | €886 |
| Inpatient Nights | Per Night | €933 | HPO | £631 | PSSRU 2019 | €739 |
| A&E Visits | Per Visit | €280 | HPO | £208 | PSSRU 2019 | €243 |

(HPO) Healthcare Pricing Office Admitted Price List 2019. Unit costs in 2019 prices. (HSE) Where necessary unit costs were inflated using the health component of the consumer price index from the Central Statistics Office. (PSSRU) Personal Social Services Research Unit in UK. Sterling prices were converted as appropriate using Purchasing Power Parity (PPP) index 2019 as per HIQA guidelines.
