# Supplementary material for: Anticipatory care planning for community-dwelling older adults at risk of functional decline: a feasibility cluster randomized controlled trial
Source: BMC Geriatr. 2022 May 25;22:452. doi: 10.1186/s12877-022-03128-x (PMC9131621; doi:10.1186/s12877-022-03128-x)
Supplement: Supplementary file 5 — Additional file 5: Supplementary Table 1. Comparisons of outcomes between intervention and usual care at 10 weeks and 6 months, based upon complete case using a multilevel model to calculate difference in mean and adjusted difference in mean. [file 12877_2022_3128_MOESM5_ESM.docx]

Supplementary Table 1: Comparisons of outcomes between intervention and usual care at 10 weeks and 6 months, based upon complete case using a multilevel model to calculate difference in mean and adjusted difference in mean.

| Outcome | Intervention | | | | | | | Usual care | | | | | | | Diff in mean^1^  (95% CI) | | P | | Adjusted^2^ diff in mean (95% CI) | | P | |  |
| --- | --- | --- | --- | --- | --- | --- | --- | --- | --- | --- | --- | --- | --- | --- | --- | --- | --- | --- | --- | --- | --- | --- | --- |
|  | Baseline | |  | | Endpoint | |  | | Baseline | |  | | Endpoint | | |  | |  | |  | |  | |
|  | N | Mean (sd) | | n | | Mean (sd) | | n | | Mean (sd) | | n | | Mean (sd) |  | |  | |  | |  | |  |
| 10 week analysis | | | | | | | | | | | | | | | | | | | | | | |  |
| Primary outcomes |  |  | |  | |  | |  | |  | |  | |  |  | |  | |  | |  | |  |
| EQ-5D-5L index score | 34 | 0.72 (0.19) | | 34 | | 0.69 (0.21) | | 31 | | 0.65 (0.25) | | 28 | | 0.65 (0.31) | -0.04 (-0.16,0.08) | | 0.486 | | -0.01 (-0.09,0.07) | | 0.808 | |  |
| EQ-VAS score | 34 | 62.2 (19.9) | | 34 | | 64.9 (15.4) | | 31 | | 61.2 (15.6) | | 28 | | 65.9 (18.2) | -1.4 (-8.0,5.3) | | 0.687 | | -1.4 (-8.5,5.6) | | 0.692 | |  |
| CES-D | 34 | 9.1 (9.1) | | 34 | | 9.3 (9.6) | | 31 | | 10.6 (9.1) | | 28 | | 8.5 (8.8) | 2.1 (-1.2,5.4) | | 0.202 | | 2.4 (-0.9,5.6) | | 0.157 | |  |
| Secondary outcomes |  |  | |  | |  | |  | |  | |  | |  |  | |  | |  | |  | |  |
| PACIC | 34 | 2.0 (0.5) | | 34 | | 2.1 (0.8) | | 31 | | 2.1 (0.7) | | 28 | | 1.8 (0.5) | 0.3 (-0.1,0.7) | | 0.115 | | 0.4 (0.1,0.6) | | 0.015 | |  |
| KATZ Index | 34 | 5.4 (1.0) | | 34 | | 5.3 (0.8) | | 31 | | 5.1 (1.3) | | 28 | | 5.2 (1.1) | -0.1 (-0.4,0.1) | | 0.224 | | -0.2 (-0.4,0.0) | | 0.063 | |  |
| GAD-7 | 34 | 2.3 (3.2) | | 34 | | 2.6 (3.2) | | 31 | | 2.5 (2.8) | | 28 | | 2.4 (2.7) | 0.5 (-1.0,2.1) | | 0.479 | | 0.1 (-1.1,1.2) | | 0.921 | |  |
| MOS Social Support Score | 34 | 4.2 (0.8) | | 34 | | 4.5 (0.5) | | 31 | | 4.3 (0.8) | | 28 | | 4.2 (1.1) | 0.4 (0.1,0.7) | | 0.022 | | 0.5 (0.2,0.8) | | 0.003 | |  |
| 6 month analysis | | | | | | | | | | | | | | | | | | | | | | |  |
| Primary outcomes |  |  | |  | |  | |  | |  | |  | |  |  | |  | |  | |  | |  |
| EQ-5D-5L index score | 34 | 0.72 (0.19) | | 34 | | 0.65 (0.27) | | 31 | | 0.65 (0.25) | | 26 | | 0.67 (0.28) | -0.08 (-0.19,0.04) | | 0.202 | | -0.07 (-0.16,0.03) | | 0.17 | |  |
| EQ-VAS score | 34 | 62.2 (19.9) | | 34 | | 63.1 (20.2) | | 31 | | 61.2 (15.6) | | 26 | | 66.9 (12.3) | -3.9 (-11.7,3.9) | | 0.326 | | -5.1 (-12.9,2.8) | | 0.205 | |  |
| CES-D | 34 | 9.1 (9.1) | | 34 | | 9.6 (7.1) | | 31 | | 10.6 (9.1) | | 26 | | 8.4 (7.7) | 1.6 (-1.5,4.7) | | 0.306 | | 1.2 (-1.7,4.1) | | 0.431 | |  |
| Secondary outcomes |  |  | |  | |  | |  | |  | |  | |  |  | |  | |  | |  | |  |
| PACIC | 34 | 2.0 (0.5) | | 34 | | 2.1 (0.9) | | 31 | | 2.1 (0.7) | | 26 | | 1.8 (0.8) | 0.5 (0.1,0.9) | | 0.022 | | 0.4 (-0.0,0.8) | | 0.061 | |  |
| KATZ Index | 34 | 5.4 (1.0) | | 34 | | 5.3 (1.2) | | 31 | | 5.1 (1.3) | | 26 | | 5.2 (1.2) | -0.2 (-0.6,0.1) | | 0.201 | | -0.3 (-0.6,0.1) | | 0.103 | |  |
| GAD-7 | 34 | 2.3 (3.2) | | 34 | | 3.1 (3.5) | | 31 | | 2.5 (2.8) | | 26 | | 2.3 (3.3) | 0.8 (-0.8,2.4) | | 0.307 | | 0.3 (-1.2,1.9) | | 0.683 | |  |
| MOS Social Support Score | 34 | 4.2 (0.8) | | 34 | | 4.4 (0.7) | | 31 | | 4.3 (0.8) | | 26 | | 3.7 (1.1) | 0.7 (0.3,1.1) | | 0.001 | | 0.8 (0.5,1.2) | | <0.001 | |  |

^1^ Using ANCOVA, and adjusting for clustering using a multilevel model with a random intercept by practice.

^2^ Same as ^1^ but additionally adjusting for gender, age, region, living arrangements (alone/couple/extended family/assisted living), carer (yes/no) and cared for (yes/no).
