# Supplementary material for: Anticipatory care planning for community-dwelling older adults at risk of functional decline: a feasibility cluster randomized controlled trial
Source: BMC Geriatr. 2022 May 25;22:452. doi: 10.1186/s12877-022-03128-x (PMC9131621; doi:10.1186/s12877-022-03128-x)
Supplement: Supplementary file 6 — Additional file 6: Supplementary Table 2. Comparisons of outcomes between intervention and usual care at 10 weeks and 6 months, based upon multiple imputation. [file 12877_2022_3128_MOESM6_ESM.docx]

Supplementary Table 2: Comparisons of outcomes between intervention and usual care at 10 weeks and 6 months, based upon multiple imputation.

| Outcome | Intervention | | | | | | Usual care | | | | | | Diff in mean^1^  (95% CI) | P | Adjusted^2^ diff in mean (95% CI) | P |
| --- | --- | --- | --- | --- | --- | --- | --- | --- | --- | --- | --- | --- | --- | --- | --- | --- |
|  | Baseline | |  | Endpoint | |  | Baseline | |  | | Endpoint | |  |  |  |  |
|  | N | Mean (sd) | | n | Mean (sd) | | n | Mean (sd) | | n | | Mean (sd) |  |  |  |  |
| 10 week analysis | | | | | | | | | | | | | | | | |
| Primary outcomes |  |  | |  |  | |  |  | |  | |  |  |  |  |  |
| EQ-5D-5L index score | 34 | 0.72 (0.19) | | 34 | 0.69 (0.21) | | 31 | 0.65 (0.25) | | 28 | | 0.65 (0.31) | -0.03 (-0.19,0.13) | 0.645 | -0.01 (-0.08,0.06) | 0.706 |
| EQ-VAS score | 34 | 62.2 (19.9) | | 34 | 64.9 (15.4) | | 31 | 61.2 (15.6) | | 28 | | 65.9 (18.2) | -1.5 (-9.2,6.3) | 0.652 | -1.4 (-9.3,6.5) | 0.669 |
| CES-D | 34 | 9.1 (9.1) | | 34 | 9.3 (9.6) | | 31 | 10.6 (9.1) | | 28 | | 8.5 (8.8) | 2.1 (-1.5,5.6) | 0.193 | 2.4 (-0.9,5.7) | 0.126 |
| Secondary outcomes |  |  | |  |  | |  |  | |  | |  |  |  |  |  |
| PACIC | 34 | 2.0 (0.5) | | 34 | 2.1 (0.8) | | 31 | 2.1 (0.7) | | 28 | | 1.8 (0.5) | 0.3 (-0.2,0.9) | 0.203 | 0.4 (-0.0,0.7) | 0.065 |
| KATZ Index | 34 | 5.4 (1.0) | | 34 | 5.3 (0.8) | | 31 | 5.1 (1.3) | | 28 | | 5.2 (1.1) | -0.1 (-0.4,0.1) | 0.263 | -0.2 (-0.5,0.1) | 0.133 |
| GAD-7 | 34 | 2.3 (3.2) | | 34 | 2.6 (3.2) | | 31 | 2.5 (2.8) | | 28 | | 2.4 (2.7) | 0.6 (-1.3,2.5) | 0.474 | 0.1 (-1.6,1.7) | 0.927 |
| MOS Social Support Score | 34 | 4.2 (0.8) | | 34 | 4.5 (0.5) | | 31 | 4.3 (0.8) | | 28 | | 4.2 (1.1) | 0.4 (-0.1,0.9) | 0.102 | 0.5 (-0.0,1.0) | 0.063 |
| 6 month analysis | | | | | | | | | | | | | | | | |
| Primary outcomes |  |  | |  |  | |  |  | |  | |  |  |  |  |  |
| EQ-5D-5L index score | 34 | 0.72 (0.19) | | 34 | 0.65 (0.27) | | 31 | 0.65 (0.25) | | 26 | | 0.67 (0.28) | -0.07 (-0.23,0.09) | 0.315 | -0.07 (-0.19,0.05) | 0.191 |
| EQ-VAS score | 34 | 62.2 (19.9) | | 34 | 63.1 (20.2) | | 31 | 61.2 (15.6) | | 26 | | 66.9 (12.3) | -3.5 (-14.9,7.9) | 0.46 | -5.3 (-17.7,7.1) | 0.325 |
| CES-D | 34 | 9.1 (9.1) | | 34 | 9.6 (7.1) | | 31 | 10.6 (9.1) | | 26 | | 8.4 (7.7) | 1.7 (-2.7,6.1) | 0.374 | 1.1 (-2.2,4.4) | 0.413 |
| Secondary outcomes |  |  | |  |  | |  |  | |  | |  |  |  |  |  |
| PACIC | 34 | 2.0 (0.5) | | 34 | 2.1 (0.9) | | 31 | 2.1 (0.7) | | 26 | | 1.8 (0.8) | 0.5 (-0.1,1.1) | 0.081 | 0.4 (-0.1,0.8) | 0.091 |
| KATZ Index | 34 | 5.4 (1.0) | | 34 | 5.3 (1.2) | | 31 | 5.1 (1.3) | | 26 | | 5.2 (1.2) | -0.2 (-0.7,0.4) | 0.44 | -0.3 (-0.6,0.1) | 0.091 |
| GAD-7 | 34 | 2.3 (3.2) | | 34 | 3.1 (3.5) | | 31 | 2.5 (2.8) | | 26 | | 2.3 (3.3) | 1.0 (-1.0,2.9) | 0.252 | 0.3 (-1.7,2.3) | 0.711 |
| MOS Social Support Score | 34 | 4.2 (0.8) | | 34 | 4.4 (0.7) | | 31 | 4.3 (0.8) | | 26 | | 3.7 (1.1) | 0.7 (0.1,1.3) | 0.024 | 0.8 (0.4,1.3) | 0.005 |

^1^ Using ANCOVA, and adjusting for clustering using robust standard errors (with 8 practices).

^2^ Same as ^1^ but additionally adjusting for gender, age, region, living arrangements (alone/couple/extended family/assisted living), carer (yes/no) and cared for (yes/no).

Multiple imputation was based upon 10 imputed datasets. The multiple imputation model was conducted using linear regression the model contained the baseline value of each outcome, group gender, age, region, living arrangements (alone/couple/extended family/assisted living), carer (yes/no) and cared for (yes/no).
