# Supplementary material for: Anticipatory care planning for community-dwelling older adults at risk of functional decline: a feasibility cluster randomized controlled trial
Source: BMC Geriatr. 2022 May 25;22:452. doi: 10.1186/s12877-022-03128-x (PMC9131621; doi:10.1186/s12877-022-03128-x)
Supplement: Supplementary file 7 — Additional file 7: Supplementary Table 3. Medians and interquartile ranges of outcomes at 10 weeks and 6 months. [file 12877_2022_3128_MOESM7_ESM.docx]

Supplementary Table 3: Medians and interquartile ranges of outcomes at 10 weeks and 6 months.

| Outcome | Intervention | | | | | | | | | | | Usual care | | | | | | | | | | |
| --- | --- | --- | --- | --- | --- | --- | --- | --- | --- | --- | --- | --- | --- | --- | --- | --- | --- | --- | --- | --- | --- | --- |
|  | Baseline | | | |  | Endpoint | | | |  | Baseline | | | | |  | | Endpoint | | | | |
|  | N | Median | Lower  quartile | Upper  quartile | | n | Median | Lower  quartile | Upper  quartile | | n | | Median | Lower  quartile | Upper  quartile | | n | | Median | Lower  quartile | Upper  quartile |  |
| 10 week analysis | | | | | | | | | | | | | | | | | | | | | |  |
| Primary outcomes |  |  |  |  | |  |  |  |  | |  | |  |  |  | |  | |  |  |  |  |
| EQ-5D-5L index score | 34 | 0.8 | 0.6 | 0.9 | | 34 | 0.7 | 0.6 | 0.9 | | 31 | | 0.7 | 0.6 | 0.8 | | 28 | | 0.7 | 0.6 | 0.9 |  |
| EQ-VAS score | 34 | 67.5 | 50 | 80 | | 34 | 70 | 50 | 75 | | 31 | | 60 | 50 | 75 | | 28 | | 70 | 51.3 | 80 |  |
| CES-D | 34 | 6 | 4 | 10.3 | | 34 | 5.5 | 3 | 12 | | 31 | | 8 | 4 | 17 | | 28 | | 6 | 3 | 10 |  |
| Secondary outcomes |  |  |  |  | |  |  |  |  | |  | |  |  |  | |  | |  |  |  |  |
| PACIC | 34 | 1.9 | 1.6 | 2.3 | | 34 | 1.8 | 1.6 | 2.3 | | 31 | | 1.9 | 1.6 | 2.5 | | 28 | | 1.8 | 1.4 | 2.2 |  |
| KATZ Index | 34 | 6 | 5 | 6 | | 34 | 5 | 5 | 6 | | 31 | | 5 | 5 | 6 | | 28 | | 5 | 5 | 6 |  |
| GAD-7 | 34 | 1 | 0 | 3.3 | | 34 | 1 | 0 | 4.3 | | 31 | | 2 | 0 | 4 | | 28 | | 1.5 | 0 | 3.8 |  |
| MOS Social Support Score | 34 | 4.4 | 3.7 | 4.9 | | 34 | 4.7 | 4.4 | 4.9 | | 31 | | 4.5 | 3.7 | 5 | | 28 | | 4.8 | 4 | 5 |  |
| 6 month analysis | | | | | | | | | | | | | | | | | | | | | |  |
| Primary outcomes |  |  |  |  | |  |  |  |  | |  | |  |  |  | |  | |  |  |  |  |
| EQ-5D-5L index score | 34 | 0.8 | 0.6 | 0.9 | | 34 | 0.7 | 0.5 | 0.9 | | 31 | | 0.7 | 0.6 | 0.8 | | 26 | | 0.7 | 0.4 | 0.9 |  |
| EQ-VAS score | 34 | 67.5 | 50 | 80 | | 34 | 65 | 50 | 75 | | 31 | | 60 | 50 | 75 | | 26 | | 70 | 60 | 70 |  |
| CES-D | 34 | 6 | 4 | 10.3 | | 34 | 8.5 | 4 | 14.3 | | 31 | | 8 | 4 | 17 | | 26 | | 5 | 3 | 14.3 |  |
| Secondary outcomes |  |  |  |  | |  |  |  |  | |  | |  |  |  | |  | |  |  |  |  |
| PACIC | 34 | 1.9 | 1.6 | 2.3 | | 34 | 1.8 | 1.4 | 2.7 | | 31 | | 1.9 | 1.6 | 2.5 | | 26 | | 1.5 | 1.4 | 1.9 |  |
| KATZ Index | 34 | 6 | 5 | 6 | | 34 | 6 | 5 | 6 | | 31 | | 5 | 5 | 6 | | 26 | | 5.5 | 5 | 6 |  |
| GAD-7 | 34 | 1 | 0 | 3.3 | | 34 | 2 | 0.8 | 3.5 | | 31 | | 2 | 0 | 4 | | 26 | | 1 | 0 | 4 |  |
| MOS Social Support Score | 34 | 4.4 | 3.7 | 4.9 | | 34 | 4.7 | 4.2 | 4.9 | | 31 | | 4.5 | 3.7 | 5 | | 26 | | 3.7 | 3.2 | 4.7 |  |
