# Supplementary material for: Anticipatory care planning for community-dwelling older adults at risk of functional decline: a feasibility cluster randomized controlled trial
Source: BMC Geriatr. 2022 May 25;22:452. doi: 10.1186/s12877-022-03128-x (PMC9131621; doi:10.1186/s12877-022-03128-x)
Supplement: Supplementary file 8 — Additional file 8: Supplementary Table 4. ICCs for primary outcomes [file 12877_2022_3128_MOESM8_ESM.docx]

Supplementary Table 4: ICCs for primary outcomes

|  | Baseline  ICC^1^ | | | 10 week  ICC1 | | | 6 month  ICC^1^ | | |
| --- | --- | --- | --- | --- | --- | --- | --- | --- | --- |
|  | ICC | Lower  95%CI | Upper  95%CI | ICC | Lower  95%CI | Upper  95%CI | ICC | Lower  95%CI | Upper  95%CI |
| EQ-5D-5L | 0.08 | 0 | 0.27 | 0.06 | 0 | 0.25 | 0 | 0 | 0.15 |
| EQ-VAS | 0.08 | 0 | 0.28 | 0 | 0 | 0.15 | 0 | 0 | 0.15 |
| CES-D | 0 | 0 | 0.14 | 0 | 0 | 0.15 | 0 | 0 | 0.15 |
| PACIC | 0.12 | 0 | 0.34 | 0.13 | 0 | 0.38 | 0.03 | 0 | 0.2 |
| KATZ Index | 0.05 | 0 | 0.23 | 0 | 0 | 0.15 | 0.05 | 0 | 0.25 |
| GAD-7 | 0 | 0 | 0.14 | 0.08 | 0 | 0.29 | 0 | 0 | 0.15 |
| MOS Social Support Score | 0.14 | 0 | 0.37 | 0.23 | 0 | 0.52 | 0.25 | 0 | 0.56 |

^1^ICC and confidence interval based upon random effects ANOVA using all 8 practices (calculating using Stata loneway routine).
